# Borderline Brenner tumor of the ovary with mucinous metaplasia: A case report

**DOI:** 10.1002/ccr3.7967

**Published:** 2023-09-27

**Authors:** Zahra Shiravani, Samaneh Bahrami, Fateme Sadat Najib, Seyed Mohammad Amin Alavi

**Affiliations:** ^1^ Department of Obstetrics and Gynecology Division of Oncology Gynecology School of Medicine Shiraz University of Medical Sciences Shiraz Iran; ^2^ Maternal‐Fetal Medicine Research Center Shiraz University of Medical Sciences Shiraz Iran; ^3^ Department of Obstetrics and Gynecology Division of Perinatology Imam Khomeini Hospital Ahvaz Jundishapur University of Medical Sciences Ahvaz Iran; ^4^ Infertility Research Center Shiraz University of Medical Sciences Shiraz Iran; ^5^ Faculty of Medicine Ahvaz Jundishapur University of Medical Sciences Ahvaz Iran

**Keywords:** atypical Brenner tumor, epithelial ovarian neoplasm, mucinous metaplasia, salpingo‐oophorectomy

## Abstract

**Key Clinical Message:**

To date, there have been disparate reports regarding borderline forms of Brenner tumors, while research on concomitant mucinous proliferation is even more irregular; however, it has been observed that proper diagnosis and treatment have proven to have a favorable prognosis on the aforementioned tumor.

**Abstract:**

Brenner tumor is a rare epithelial ovarian neoplasm responsible for 2%–3% of all ovarian tumors. These tumors are usually asymptomatic and can usually be found incidentally in pathological studies. They can also manifest themselves as abdominal pain or abnormal uterine bleeding. A 41‐year‐old female with a history of anemia referred to the hospital suffering from abdominal pain for at least 1 month before the referral period. Physical examination revealed a mobile palpable mass in the left lower abdomen. Ultrasound and pelvic computed tomography scan (CT scan) revealed a left ovarian complex cyst. Left salpingo‐oophorectomy was performed on the patient, and the cyst was removed. Pathological findings revealed an atypical proliferative Brenner tumor with mucinous metaplasia. To date, there have been disparate reports regarding borderline forms of Brenner tumors, while research on concomitant mucinous proliferation is even more irregular; however, it has been observed that proper diagnosis and treatment have proven to have a favorable prognosis on the aforementioned tumor.

## Graphical abstract

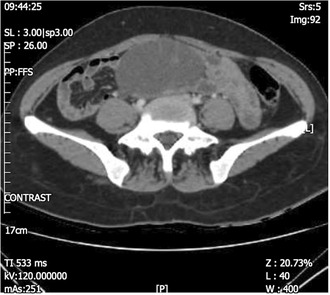

## 1 INTRODUCTION

MacNaughton‐Jones reported the first observed case of a Brenner tumor in 1898. Nine years later, Fritz Brenner described the tumor that carries his name. A Brenner tumor originally arises from the follicular epithelium and comprises ovarian transitional cells enclosed by fibrous tissue.^1^, ^2^ Based on the World Health Organization (WHO) classification, Brenner tumors are classified as benign, borderline, and malignant.^3^ Brenner tumors are benign lesions, of which only 1% are malignant.^4^

Brenner tumors are generally asymptomatic and are observed incidentally; however, larger tumors might manifest themselves as pelvic mass compounded by severe pain.^5^ The Brenner tumor staging system follows that of malignant ovarian tumors; however, clinicians in practice often fail to utilize the staging system. Generally, the preferred therapeutic approach for borderline Brenner tumors is surgical resection, as no therapy in addition to surgery is needed. It is of note that there is a favorable prognosis for borderline Brenner tumors.^6^

In the current study, the authors present a rare case of borderline ovarian Brenner tumor with mucinous metaplasia, which was treated surgically.

## 2 CASE PRESENTATION

**FIGURE 1:**
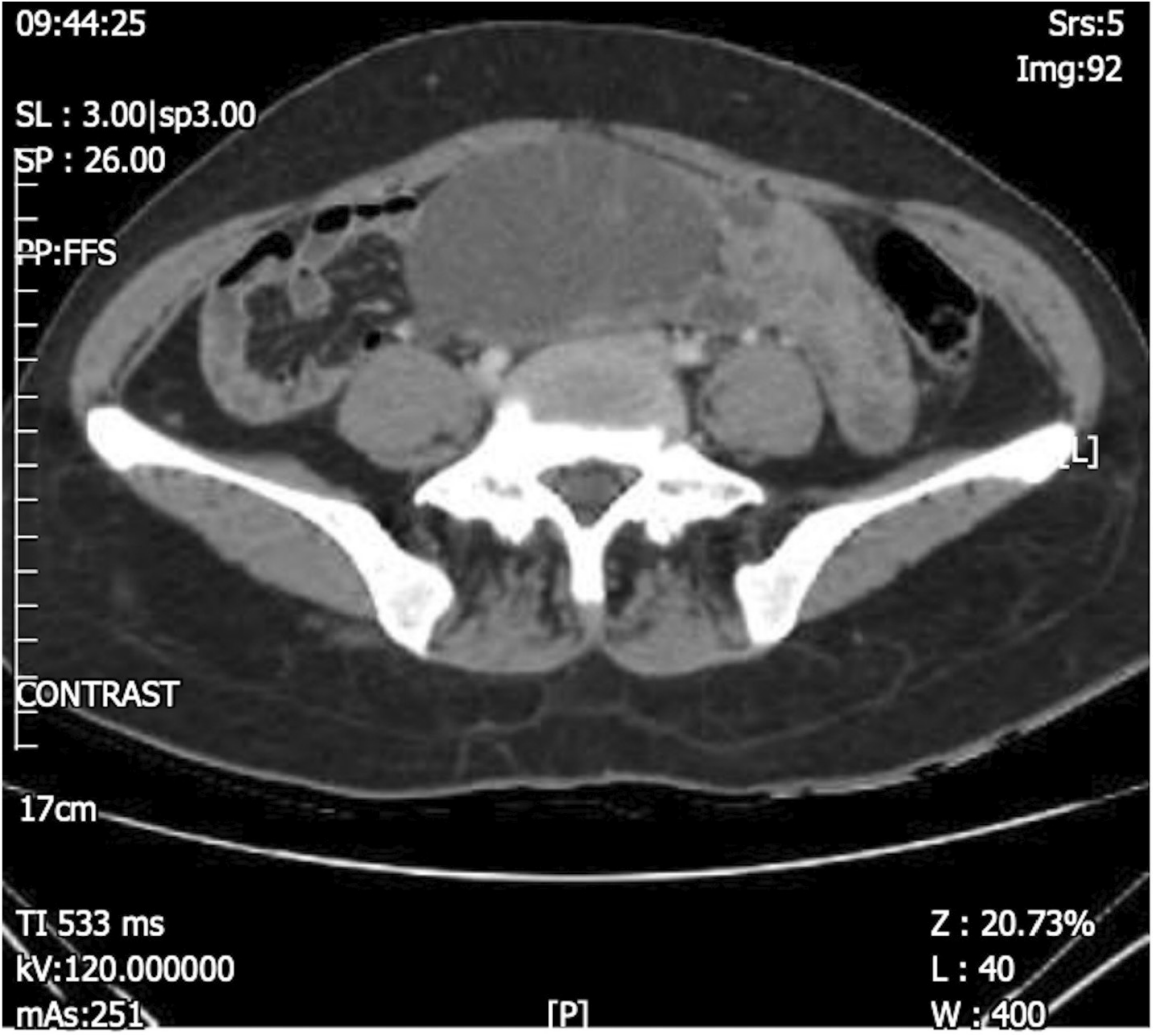
Abdominopelvic computed tomography (CT), axial, with intravenous and oral contrast, revealed a large cystic mass arising from the left adnexa with several thin enhancing septa highly suggestive of ovarian cystadenocarcinoma.

**FIGURE 2:**
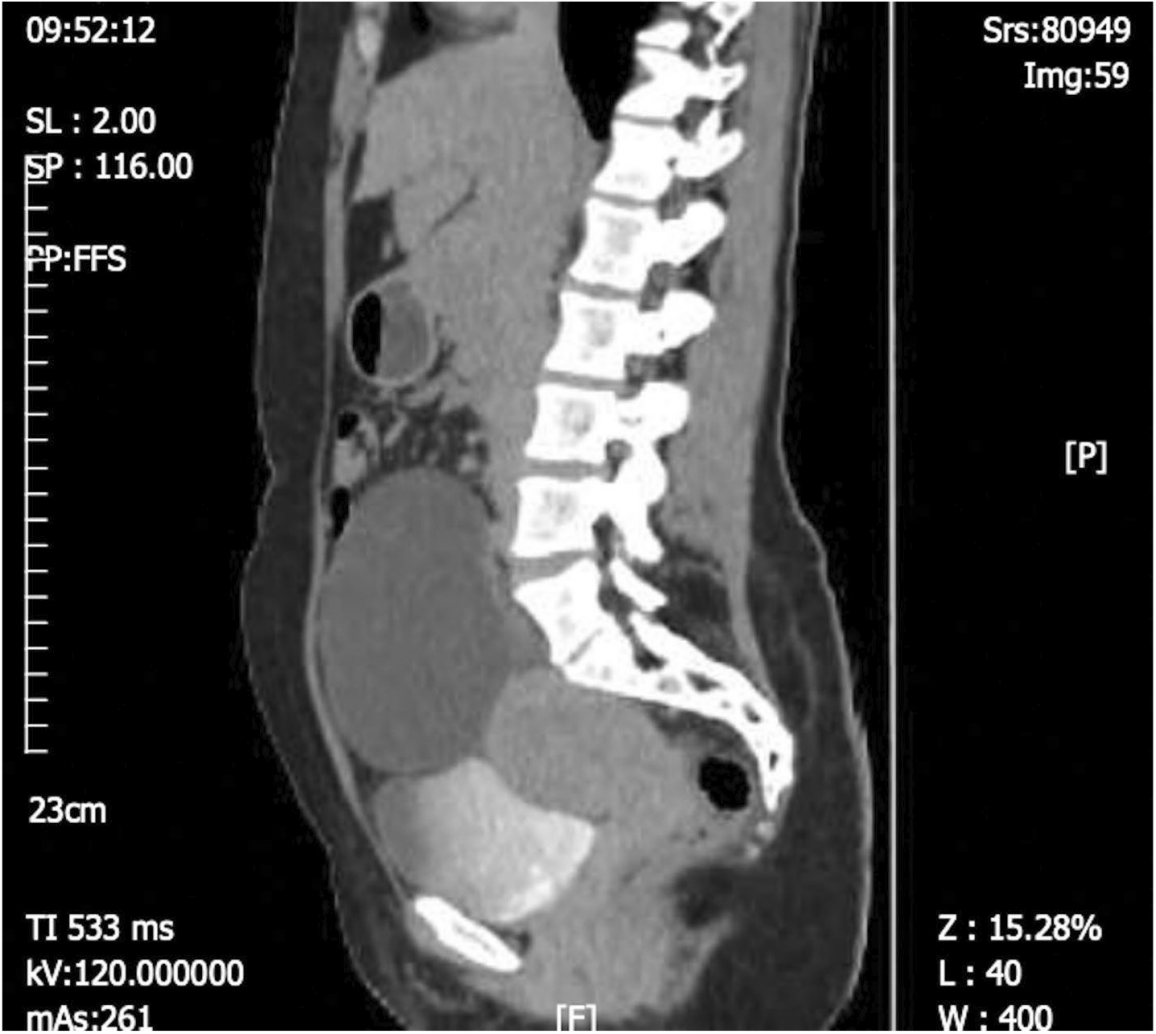
Abdominopelvic computed tomography (CT), sagittal, with intravenous and oral contrast, revealed a large cystic mass arising from the left adnexa with several thin enhancing septa highly suggestive of ovarian cystadenocarcinoma.

A 41‐year‐old G3P3 female with a history of anemia referred to the hospital suffering from abdominal pain with an onset at least 1 month before the referral period. The pain was chronic, dull, and had had a gradual onset. The patient's medical history was unremarkable, with no prior history of surgery. The patient's most recent pap smear (6 months before referral) showed nothing remarkable. Physical examination revealed a mobile palpable mass in the left lower abdomen. A vaginal examination further revealed a mass in the pelvic region; the uterus was 8 cm with normal consistency. The speculum examination showed nothing noteworthy. Abdominal and pelvic ultrasound examination and computed tomography (CT) scan revealed a 15 × 10 cm left ovarian thick wall complex cyst, suggestive of an ovarian tumor (Figures 1 and 2). Tumor markers such as cancer antigen 125 (CA125), carcinoembryonic antigen (CEA), and CA19‐9 were all within the normal range. Complete Blood Count differential count (CBC diff), liver function test, blood glucose, and kidney function test were all consistent with normal values.

**FIGURE 3:**
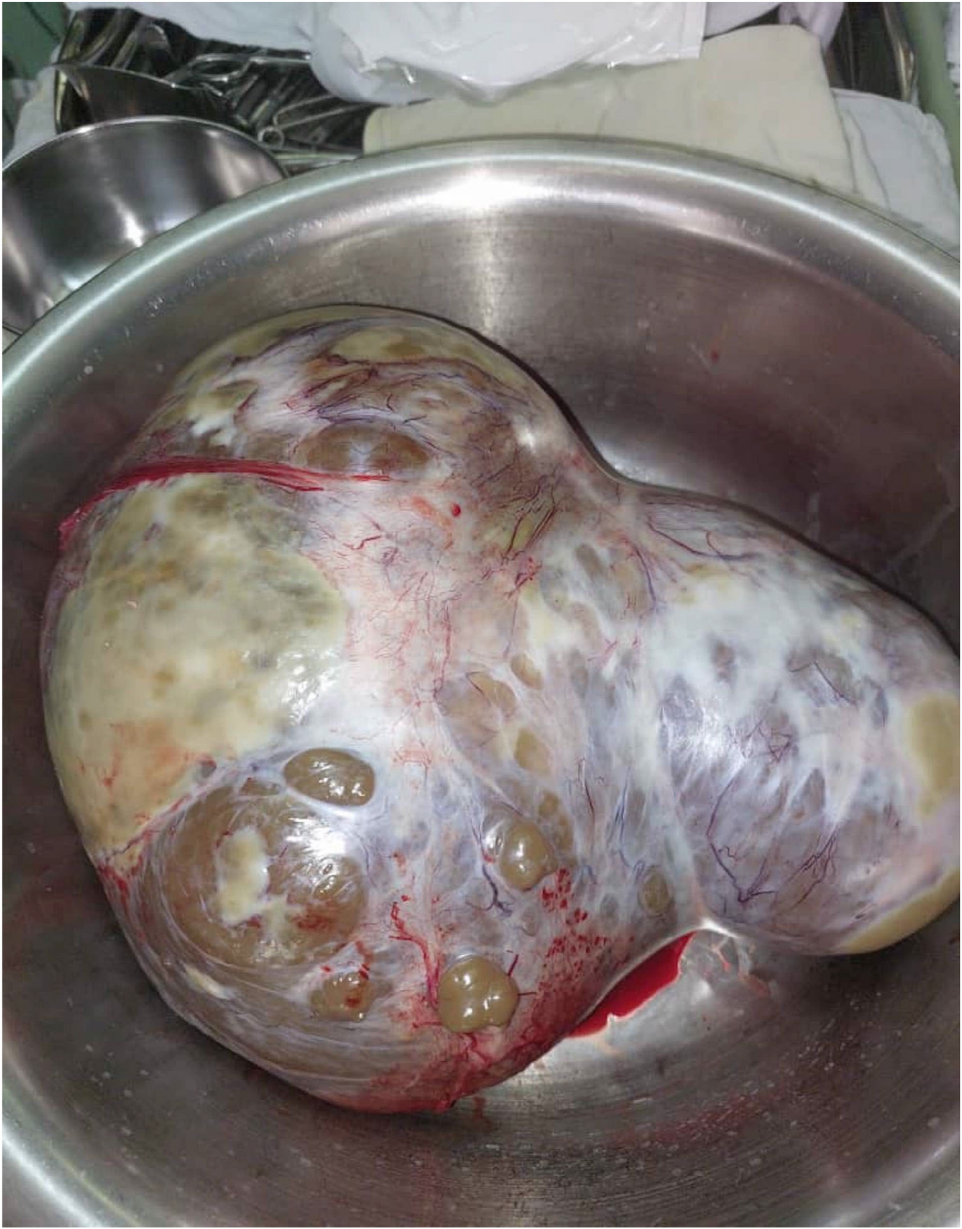
A gross pathologic examination reported a creamy‐brown color intact solid cystic mass with 1 cm wall thickness.

**FIGURE 4:**
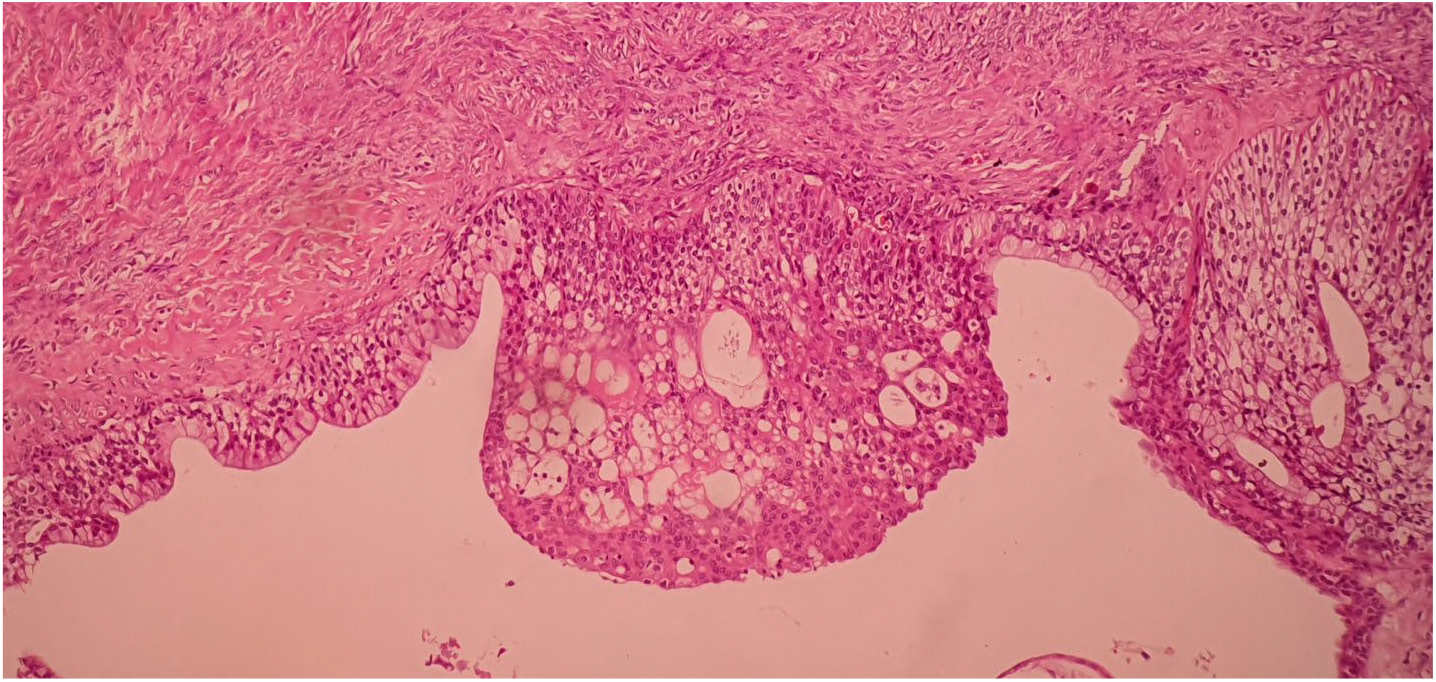
Hematoxylin and eosin stain (100×) of the surgical specimen demonstrating borderline Brenner tumor with mucinous metaplasia.

**FIGURE 5:**
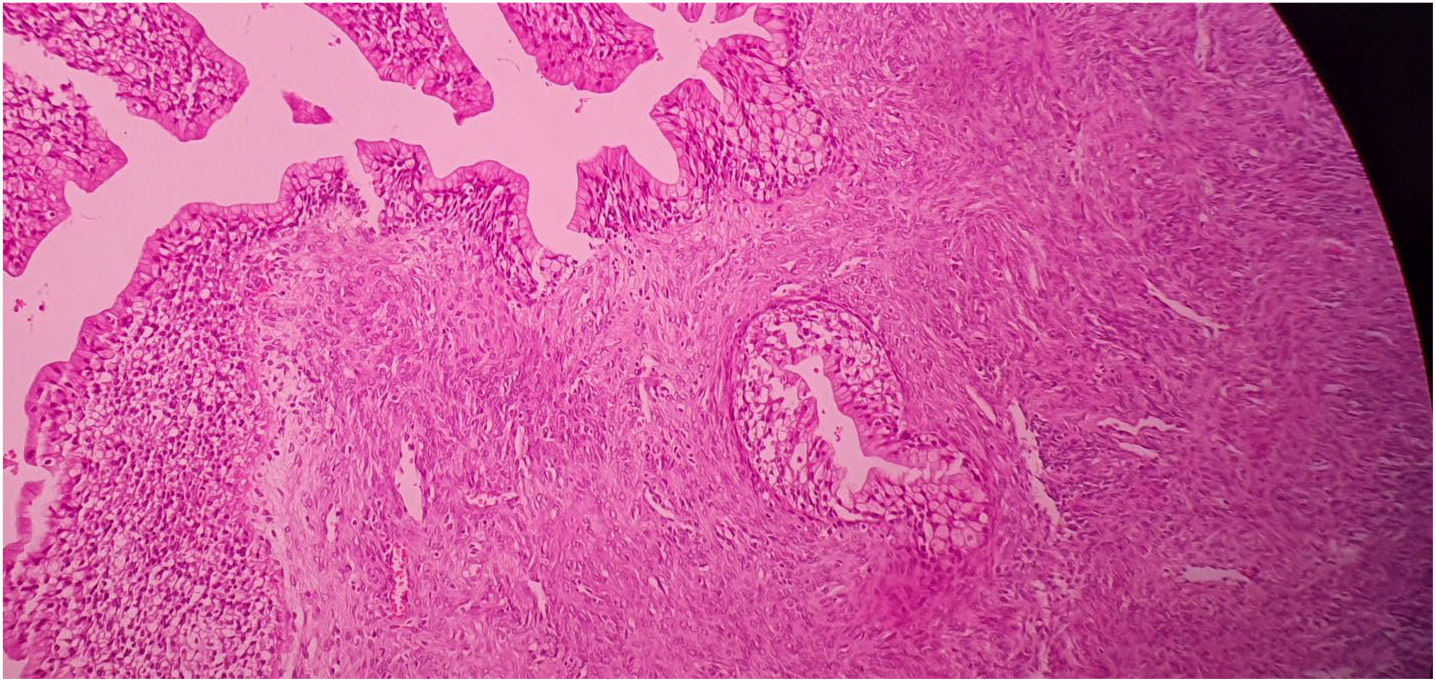
Hematoxylin and eosin stain (100×) of the surgical specimen demonstrating mucinous metaplasia.

**FIGURE 6:**
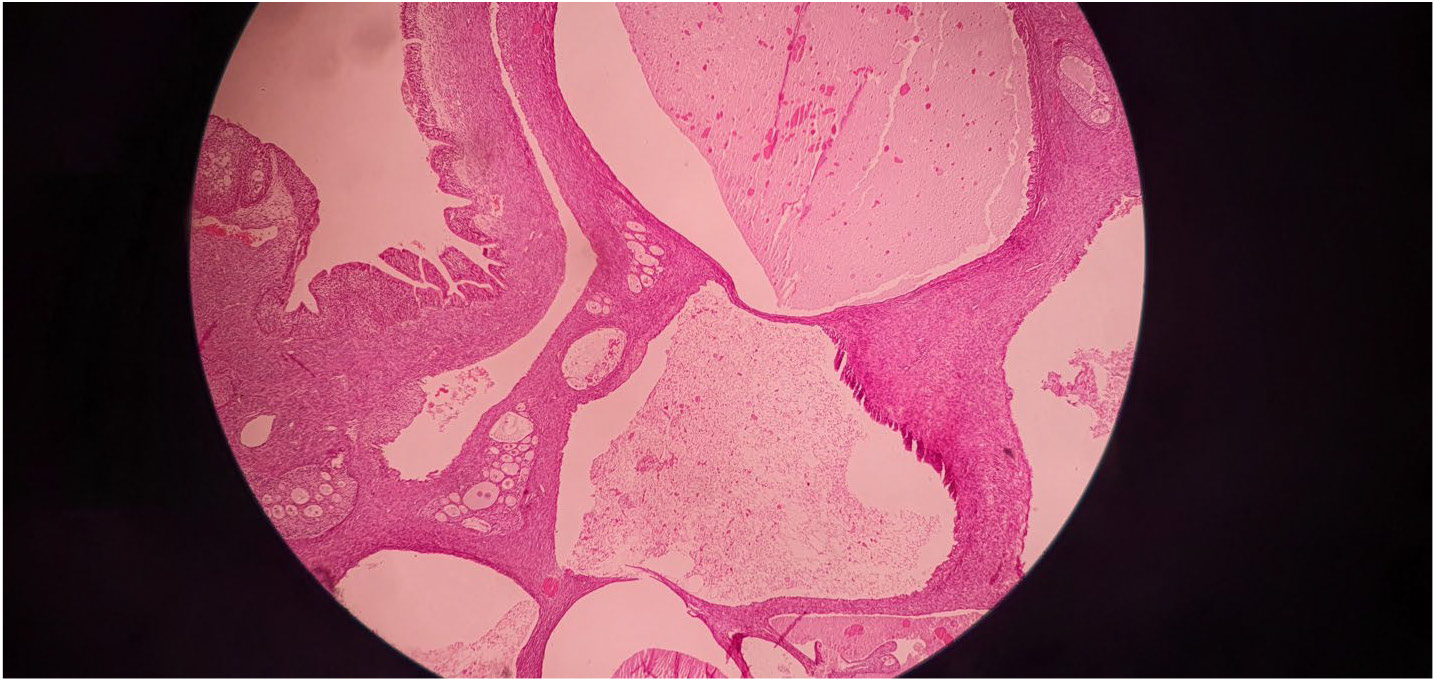
Hematoxylin and eosin stain (50×) of the surgical specimen demonstrating multiple cystic spaces lined by metaplastic mucinous epithelium and filled with mucin material.

**FIGURE 7:**
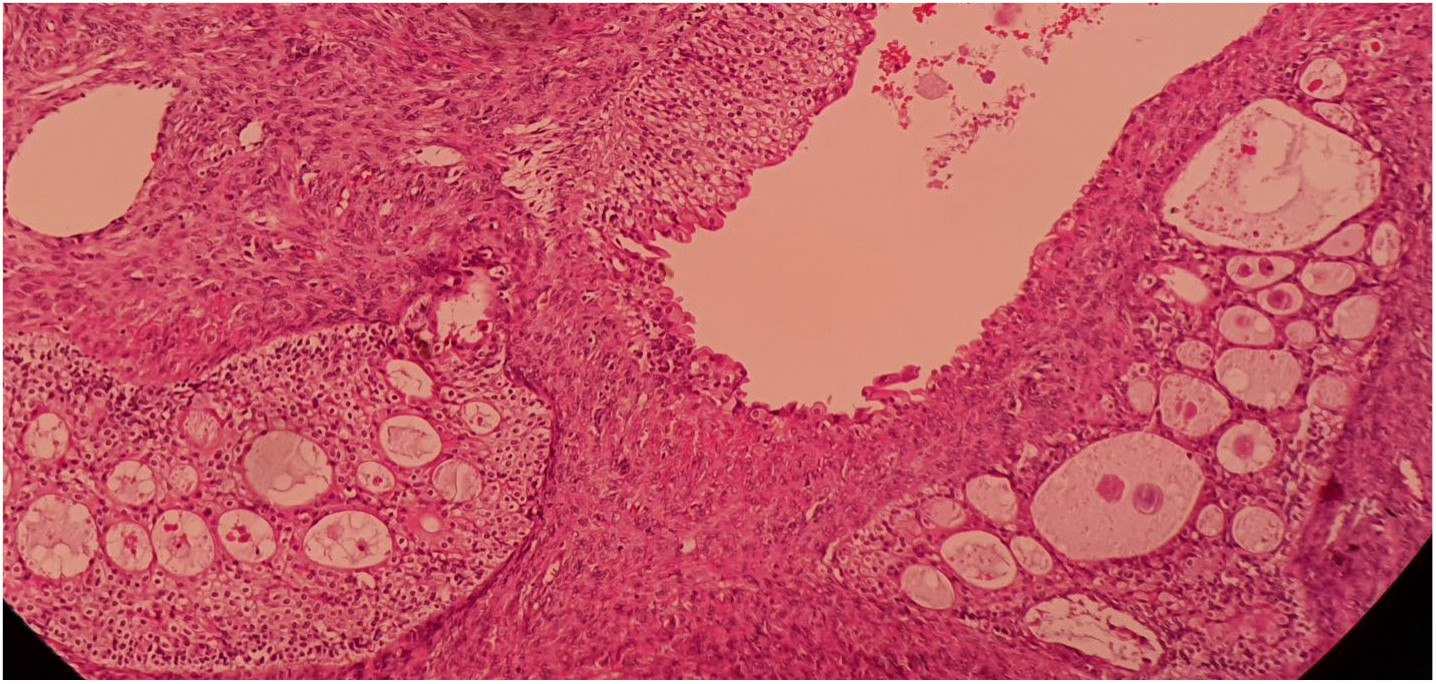
Hematoxylin and eosin stain (100×) of the surgical specimen demonstrating nests composed of transitional‐type epithelium and cystic spaces.

Based on the impression of cystadenocarcinoma as per the abdominopelvic CT findings, the patient underwent laparotomy, in which the surgical exploration revealed a multi‐lobulated solid ovarian tumor on the left side. At the same time, the uterus, right ovary, and both fallopian tubes were all normal. The peritoneal wash was collected and forwarded for cytology, which in itself demonstrated a rich cellularity composed of many groups of mesothelial cells and inflammatory cells. A left salpingo‐oophorectomy was performed, and the sample was sent for pathological examination. A gross pathologic examination reported a creamy‐brown intact solid cystic mass with 1 cm wall thickness (Figure 3). Histologic findings revealed a Stage 1 borderline Brenner tumor of the left ovary with no capsular surface involvement (Figures 4, 5, 6, 7). Microscopic examination reported cysts and nests of dense fibrotic stroma. The nests were composed of transitional‐type epithelium with mucinous metaplasia. Mild atypia and mitotic figures were present. Cystic spaces were lined by metaplastic mucinous epithelium and filled with mucin material. Pathological findings demonstrated an atypical proliferative Brenner tumor with mucinous metaplasia. The postoperative period was uneventful, and the patient was discharged with outpatient care. Following the patient's discharge, evaluations were carried out every 4 months for 2 years, in which the patient underwent a pelvic physical examination; in addition, ultrasound examinations were also carried out, revealing that the patient had no subsequent problems in the pelvic region.

## 3 DISCUSSION

Brenner tumors are rare, usually benign, small, and unilateral, of which fewer than 5% are borderline or proliferative.^7^, ^8^ Histologically, borderline Brenner tumors are similar to the low‐grade papillary urothelial neoplasms, in which the characteristics include uniformity, elongation of nuclei, and the presence of fine chromatin and prominent nucleoli; however, in some cases, there is mucinous or squamous metaplasia and moderate to severe atypia.^2^ Approximately 16% of Brenner tumors are associated with mucinous tumors. It is postulated that a significant correlation exists between mucinous and transitional cell neoplasms, which may offer invaluable insights into the etiology and advancement of ovarian mucinous epithelial malignancies.^9^

Brenner tumors consist of nests of transitional/urothelial‐type epithelium, which are surrounded by a dense fibromatous stroma.^10^ They are believed to originate from the ovarian surface epithelium through a process of transitional‐type metaplasia. However, modern theories suggest that Brenner tumors may originate from the epithelium of the fallopian tube after having undergone transitional cell metaplasia and then spread to the ovaries.^11^

Borderline Brenner tumors occur over an extensive age range, chiefly from 30 to 84 years, but are more prevalent in elderly patients, with over 80% of patients older than 50 years.^6^ Gezginç et al. analyzed 13 patients diagnosed with a Brenner tumor, whose average age was 55 years.^4^ In another study by Hemalatha et al., more than two third of the patients with a Brenner tumor were more than 40 years old,^12^ which was similar to the current case.

The most common clinical manifestations of borderline Brenner tumors are abdominal enlargement or mass, followed by abdominal pain and postmenopausal bleeding.^6^ Gezginç et al. study on 13 patients further indicated that abdominal pain is the most commonly shared symptom.^4^ However, Brenner tumors are usually asymptomatic and are often reported as an incidental pathological finding.^13^ In the current study, the patient's chief complaint was abdominal pain.

The ultrasound examination showed that the tumor size was 15 cm, which was a relatively large mass. The majority of borderline Brenner tumors are unilateral, and the size range is 5–30 cm.^6^ However, based on Montoriol et al., 11% of Brenner tumors were bilateral.^14^ The tumor observed in the reported case was a unilateral complex ovarian mass.

The levels of CEA and CA125 in the current investigation were within normal ranges, yet Brenner tumors have not been associated with a reliable tumor marker.^15^ In a study by Vali et al. on an atypical proliferative Brenner tumor case, the patient's CA125 was slightly increased.^16^ Brenner tumor might be accompanied by abnormal elevation of serum CA19‐9 level. Hence, whenever there is no logical explanation for the increase in CA19‐9, the probability of a Brenner tumor should be considered by the attending physician.^3^ However, CA 19‐9 is a highly utilized and well‐validated biomarker for the detection and diagnosis of pancreatic cancer, there is a possibility of detecting elevated CA 19‐9 levels in various forms of adenocarcinoma, particularly in advanced gastrointestinal malignancies.^17^

The Brenner tumors' radiologic studies yield non‐specific findings.^15^ Color Doppler examination reveals a small solid tumor with different echogenicity and poor blood flow. It may also manifest as a multilocular cystic tumor which has a solid ingredient in the presence of other ovarian neoplasms. Calcification is seen in the majority of cases.^18^ CT is the most effective imaging technique for revealing calcifications in Brenner tumors. The tumor is also seen as a solid or multilocular cyst.^2^

Brenner Tumors are often reported in association with mucinous neoplasms. Based on histogenesis, the aforementioned tumors are believed to originate from analogous precursors.^19^, ^20^ The pathological evaluations of the patient in the present study demonstrated that a Brenner tumor is associated with mucinous metaplasia, similar to the findings of previous studies.

The primary treatment of borderline Brenner tumors is surgical resection. Borderline Brenner tumors and malignant ovarian tumors follow similar staging, including total abdominal hysterectomy and bilateral salpingo‐oophorectomy (TAH‐BSO), omentectomy, cytology of the diaphragm, peritoneal washing, and resection of grossly visible metastases. Zheng and Heller stated that there was no significant difference in postoperative conditions over the 4 years follow‐up among both groups of patients with borderline Brenner tumors who were treated with TAH‐BSO or unilateral salpingo‐oophorectomy.^6^ However, Surgeons do not always perform borderline tumor staging.^6^

Less than 60 studies regarding borderline Brenner tumors have been published to date, and there is minimal information regarding the favorable prognosis of the aforementioned tumors alone or with mucinous neoplasms.^19^ In clinical practice, detecting tumors in the early stages is essential by employing strategies for early diagnosis and early treatment.^3^

The patient of the current study was followed up for 2 years after the surgery, in which no complication was reported. However, it is too early to determine the prognosis of the Brenner tumors. Although most Brenner tumors are benign, borderline and malignant forms should be considered.

## 4 CONCLUSION

The authors presented a borderline Brenner tumor in the left ovary, for which left salpingo‐oophorectomy was carried out as the proposed treatment. Pathological findings revealed an atypical proliferative Brenner tumor with mucinous metaplasia. To date, there have been dispersed reports of borderline forms of Brenner tumors. Moreover, studies on concomitant mucinous proliferation are even more irregular. Proper diagnosis and treatment have proven a favorable prognosis for the aforementioned tumor.

## AUTHOR CONTRIBUTIONS

**Zahra Shiravani:** Conceptualization; writing – review and editing. **Samaneh Bahrami:** Conceptualization; writing – original draft; writing – review and editing. **Fateme Sadat Najib:** Conceptualization; writing – review and editing. **Seyed Mohammad Amin Alavi:** Writing – original draft; writing – review and editing.

## FUNDING INFORMATION

No sources of funding were declared for this study.

## CONFLICT OF INTEREST STATEMENT

The authors have no conflict of interest to declare.

## ETHICAL STATEMENT

Permission from the ethics committee was not required for case reports at the institution where the research was carried out.

## CONSENT

Written informed consent for publication of the case report has been signed by the patient and is available upon request from the editors.

## Data Availability

The data supporting the findings of this study are available upon request from the corresponding author
